# Motion Parallax Improves Object Recognition in the Presence of Clutter in Simulated Prosthetic Vision

**DOI:** 10.1167/tvst.7.5.29

**Published:** 2018-10-29

**Authors:** Cheng Qiu, Kassandra R. Lee, Jae-Hyun Jung, Robert Goldstein, Eli Peli

**Affiliations:** 1 The Schepens Eye Research Institute, Massachusetts Eye and Ear, Department of Ophthalmology, Harvard Medical School, Boston, MA, USA; 2 Department of Psychology, University of Pennsylvania Philadelphia, PA, USA

**Keywords:** visual prosthesis, object recognition, motion parallax, sensory substitution device

## Abstract

**Purpose:**

Efficacy of current visual prostheses in object recognition is limited. Among various limitations to be addressed, such as low resolution and low dynamic range, here we focus on reducing the impact of background clutter on object recognition. We have proposed the use of motion parallax via head-mounted camera lateral scanning and computationally stabilizing the object of interest (OI) to support neural background decluttering. Simulations in head-mounted displays (HMD), mimicking the proposed effect, were used to test object recognition in normally sighted subjects.

**Methods:**

Images (24° field of view) were captured from multiple viewpoints and presented at a low resolution (20 × 20). All viewpoints were centered on the OI. Experimental conditions (2 × 3) included clutter (with or without) × head scanning (single viewpoint, 9 coherent viewpoints corresponding to subjects' head positions, and 9 randomly associated viewpoints). Subjects used lateral head movements to view OIs in the HMD. Each object was displayed only once for each subject.

**Results:**

The median recognition rate without clutter was 40% for all head scanning conditions. Performance with synthetic background clutter dropped to 10% in the static condition, but it was improved to 20% with the coherent and random head scanning (corrected *P* = 0.005 and *P* = 0.049, respectively).

**Conclusions:**

Background decluttering using motion parallax cues but not the coherent multiple views of the OI improved object recognition in low-resolution images. The improvement did not fully eliminate the impact of background.

**Translational Relevance:**

Motion parallax is an effective but incomplete decluttering solution for object recognition with visual prostheses.

## Introduction

An estimated 260,000 individuals in America are functionally blind.^1^ Blind individuals use mobility aids, such as long canes and guide dogs, and access text through braille and computer programs that convert text to speech. However, these tools do little in aiding search for and recognition of objects. Visual prostheses in the form of retinal implants, such as the Argus II^2^ and Alpha IMS,^3^ reportedly provide rudimentary vision to assist with daily tasks, including letter identification^2^ and shape detection, localization, and recognition.^3^ Sensory-substitution devices (SSDs), such as the BrainPort V100,^4,5^ a tongue-stimulation device, are also being developed to similarly represent objects to blind users.

Most visual prostheses and SSDs use video cameras to capture images and convert them into a format appropriate for the device. These final “images” are restricted by the limitations of the devices and the physiological interface. They often have low spatial resolution, low dynamic range (number of distinguishable gray levels), and narrow field of view (FoV). For example, Argus II has 60 electrodes (10 × 6 resolution) covering a retinal FoV of approximately 18° × 11°^6^ with very few gray levels. The BrainPort V100 and V200 devices differ in their physical controls, but both have similar technical specifications: 400 electrodes (20 × 20 resolution) with few gray levels.^4^ Their camera FoV defaults to 24° × 24°, and can vary from 48° × 48° to 2° × 2° using digital zoom. The digital zoom out further reduces image quality. With full-field images the camera auto exposure is appropriate for the whole image, but using digital zoom to simply crop a smaller portion of the image frequently results in low contrast.^7^ Such low-contrast features would be further degraded by the resolution reduction to 20 × 20. Low resolution and low dynamic range alone lead to images that may be difficult to interpret even when viewed with normal vision.^8^

These limitations further hinder a user's ability to recognize objects using these devices when the object of interest (OI) is in front of cluttered background. Even with normal vision, clutter can interact with the features of a target item, thus reducing performance in object recognition.^9^ This can be more severe with the poor image quality of prosthetic vision. The difficulty in interpreting local features impedes the separation of target signals from surroundings.^10^ Object recognition results reported with visual prostheses are often demonstrated under unrealistic conditions where only a small number of objects are presented to subjects and are placed over a highly contrasting, uniform (uncluttered) background that also reduces light reflections and shadows.^3,11,12^ These results may be overstating the device performance that can be expected in a real-world environment.

Jung et al.^10,13^ proposed a light-field–based method that separates an OI from background clutter and removes the clutter from images. Other techniques to achieve background decluttering include a video processing system based on optic flow,^14^ and methods using thermal imaging (Dagnelie G, et al. *IOVS*. 2016;57:ARVO E-Abstract 5167) or stereo cameras (Dagnelie G, et al. *IOVS*. 2017;58:ARVO E-Abstract 4686). Jung et al.^10^ further showed the effectiveness of decluttering in improving object recognition with simulated prosthetic vision. Interestingly, they also observed that when subjects were unable to recognize object images, they engaged in head rotation and lateral translation as if they were trying to separate object from background intuitively from multiple viewpoints,^10^ though this was not effective with static images. An increase in head movements was also observed in a visual acuity task with simulated prosthetic vision.^15^ These head movements suggest that normally sighted subjects were actively trying to maximize visual information as they might do when viewing objects naturally.

Users of simulated prosthetic vision reportedly benefited from an expanded effective FoV and improved depth perception by using lateral head movements.^16^ A change in the observer's viewing position may create motion parallax. This motion parallax causes different image movements for objects at different distances, creating cues that may aid in object and ground separation.^17^ A prosthetic vision device that can capture these characteristics of motion parallax may be useful. Schiller^18^ discussed the usefulness of motion parallax as a depth cue for visual prostheses. He assumed that video-based visual prostheses with head movement provide similar motion parallax effects as experienced in normal vision. However, we noted that retinal image motion in retinal prostheses with head-mounted video cameras was different than the retinal image motion under natural viewing with similar head motions.^13,19^ Naturally, compensatory eye rotations based in part on vestibulo-ocular reflex stabilize the retinal image of an OI on the fovea in motion parallax.

When using the head-mounted camera, the camera continues to be pointed generally straight during lateral head movements. It does not rotate toward the OI as the eyes do during similar head movements with normal vision. As a result, both the OI and the background move across the visual field (though not at the same speed), which diminishes the ability to separate the OI from the background. Additionally, the OI movements may carry it out of the narrow FoV of the device. Therefore, while trying to use motion parallax to facilitate object recognition in visual prostheses, it is important to stabilize the OI at the center of the image. Previous research has considered similar OI stabilization during rotational head movements to incorporate the vestibulo-ocular reflex into the prosthesis simulation^20^: eye tracking was used to display eye-contingent information with Argus II recipients. Their results indicated that the use of eye and head rotations together reduced head movements and improved the hand pointing precision of Argus II users. However, fixating during lateral head shifts (rather than rotations) requires the user to locate the OI within a complex real-life scene, a difficult task for a blind eye. We have proposed a computational solution intended to bypass this difficulty and to provide an OI-stabilizing mechanism, similar to fixation in normal vision.^13,21^ In this study, we tested the above principle using a simulation of the proposed motion parallax method and evaluating subjects' ability to separate an OI from background and to recognize objects in low-resolution images.

## General Methods and Materials

The impact of motion parallax on object recognition was measured using low-resolution (20 × 20) images of objects. To simulate the effect of our proposed system, we created a dataset of object images simulating those that would be generated by a visual prosthesis equipped with our system. Our images were of higher quality than those currently provided by most visual prostheses or SSDs. For example, our images had high dynamic range and therefore provided better image quality than the typical phosphene vision provided by current devices, such as the Argus II. We tested images of better quality to investigate the effect of motion parallax with OI-stabilization prior to trying to implement it with more degraded images. Also, this concept is not designed for current visual prostheses, but future iterations of devices, many of which are increasing their resolutions to even higher ranges than those demonstrated here.^3^

Our images were captured, either with or without background clutter, using the BrainPort V200 system, which generates simulated prosthetic images with low resolution (20 × 20) but uses full dynamic range (256 levels of gray). Images were captured from nine viewpoints with the OI maintained at the center of the camera FoV, resulting in the distant background clutter changing positions within the FoV. We conducted two experiments. In a Clutter Complexity experiment, we first evaluated how backgrounds of varying complexity levels affect object recognition in static low-resolution images simulating visual prostheses. In the main Motion Parallax experiment, where the level of background complexity for each object was selected from the Clutter Complexity experiment, normally sighted subjects used lateral head movements to explore and try to recognize the OI. Our goal was to see whether motion parallax by decluttering (separating) the background from the object improves new object recognition.

### Image Set Preparation

**Figure 1:**
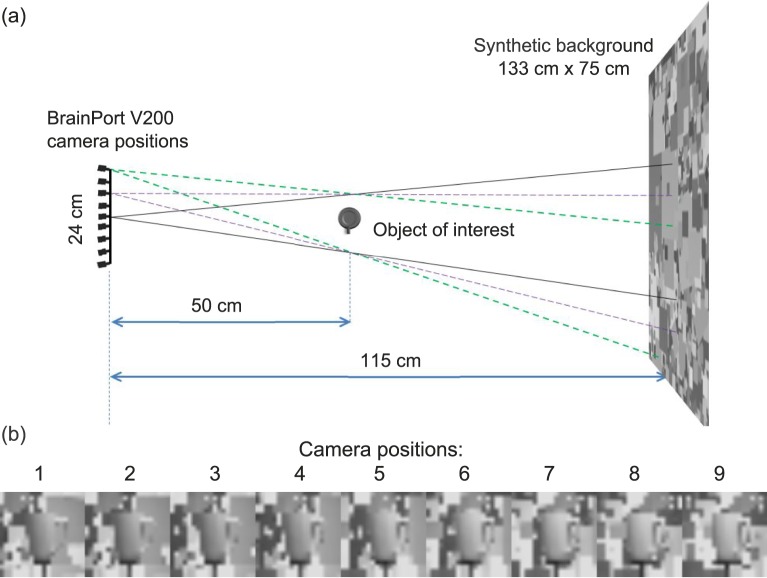
The capture of simulated prosthetic images. (a) Top view of the physical setup showing the nine camera positions on the *left*, which are 3 cm apart. For an object set 50 cm from the camera, each 3-cm lateral shift requires approximately 3° rotation to re-center the OI. For the purpose of this figure, the dead leaves background is cropped to cover the FoV of camera position rays 1, 3, and 5. (b) Captured image examples from nine lateral viewpoints from the left to the right.

A grayscale image dataset of 35 familiar objects (such as a mug, spray bottle, and teddy bear) was collected using the BrainPort V200 camera and the electrode stimulation view provided with the BrainPort to simulate low resolution of visual prosthesis. Objects were placed in front of synthetic background images at six complexity levels and photographed from nine laterally separated viewpoints (Fig. 1). The images were captured with the OI within arm's reach distances from the camera and the synthetic background images were all located 115 cm from the BrainPort V200 camera (Fig. 1a). The FoV of the camera was set at the system default of 24° × 24°. The OIs were roughly at the center of the image and covered a similar portion of image area (15°–20° in diameter). The distances between the object and the camera were 30, 50, or 70 cm, depending on the object's size (ranging from 10–30 cm), and the numbers of captured objects at these distances were 7, 12, and 16, respectively. A full list of the objects with their displayed distances and the captured images with various backgrounds can be found in the Supplementary Appendix – Object images captured with BrainPort V200 camera. The BrainPort V200 provides a high-resolution (480 × 480) camera view and a simulated prosthetic view with a true resolution of 20 × 20 that is up-sampled to 480 × 480 pixels and low-pass filtered to reduce the pixel edge artifacts (Fig. 1b). All images had a full dynamic range of 256 gray levels (as in the BrainPort simulation).

To allow rotation and lateral position shift of the BrainPort V200 camera, it was mounted on a mannequin head that was connected to a rotation optical stage located on an optical rail. Both the BrainPort camera translation and corresponding rotation to aim at the OI were calibrated to achieve imaging consistent with motion parallax in normal vision, in which the OI is fixated. The nine viewpoints provided reasonably smooth transitions when displayed in response to subjects' head movements. The lateral distance between neighboring capture positions was 3 cm, providing an overall shift of 12 cm on either side from the central viewpoint. The rotation re-centered the OI following the lateral shift. For an object placed 50 cm from the camera, the angular rotation between each viewpoint was about 3°. For objects placed 30 cm from the camera, angular change between consecutive positions was between 4° and 5°, and for objects at 70-cm distance, angular change was 2° to 2.5°.

**Figure 2:**
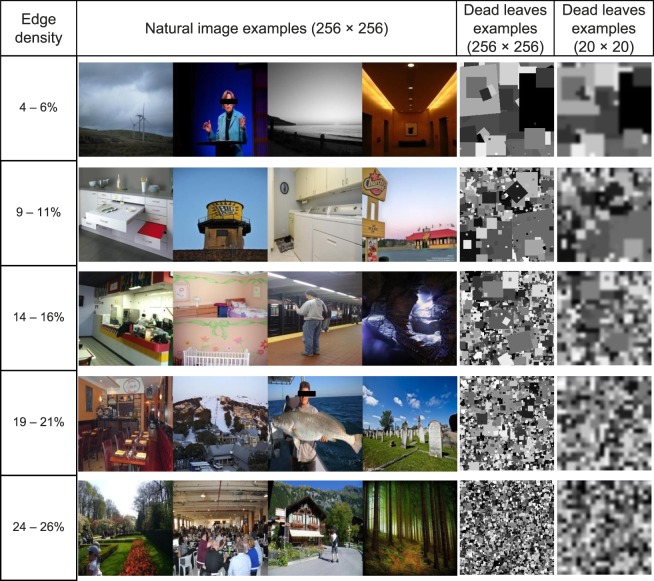
Natural image examples with various ranges of edge densities and the corresponding examples of the dead leaves backgrounds in high and low resolutions. The low-resolution images of dead leaves (20 × 20) are low-pass filtered, as in the BrainPort simulation, to reduce pixel edge artifacts.

Schematic images generated with the dead leaves model mimicking natural image statistics^22^ were used as background stimuli displayed on a large television screen (that covers the entire FoV from each viewpoint). They represented natural scene clutter while permitting controlled complexity. To quantify the background complexity, we first analyzed the distribution of edge density in 20,050 natural images (with a resolution of 256 × 256) from the MIT Places 2 dataset.^23^ For each image, edge pixels were found using the Sobel method with a fixed threshold (0.02), and then the number of edge pixels divided by the total number of image pixels was calculated as edge density.^9,24,25^ Figure 2 shows examples of the natural images at various edge density levels with corresponding examples of dead leaves images matched in edge density. Six background complexity levels were selected to cover a range of natural scene clutter, including the edge density level at 0% (blank), 5%, 10%, 15%, 20%, and 25%. Given the limited display resolution (20 × 20), clutter complexity may appear similar when edge density of the original high-resolution images was 19% to 21% and 24% to 26% (Fig. 2 last column). A set of 40 dead leaves stimuli at the television resolution (1920 × 1080 pixels) were randomly generated and verified for each of the five complexity levels with clutter.

Two experiments were conducted using the captured low-resolution images. The purpose of the first experiment was to establish recognition of new (previously unseen) objects as a function of background clutter complexity using static images. The crowdsourcing results from the first experiment, henceforth referred to as the Clutter Complexity experiment guided the selection of background complexity for our second experiment. The Motion Parallax experiment studied the impact of motion parallax on object recognition in clutter. The Massachusetts Eye and Ear Human Studies Committee, Boston, MA approved all experiments and procedures. The Committee exempted the crowdsourcing Clutter Complexity experiment. All participants in the Motion Parallax experiment signed a written informed consent before starting the experiment. All research adhered to the tenets of the Declaration of Helsinki.

## Clutter Complexity Experiment

### Crowdsourcing of Object Recognition as a Function of Background Clutter Complexity

**Figure 3:**
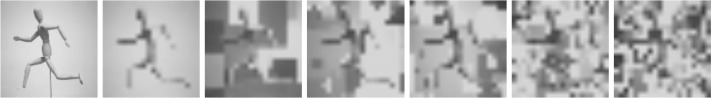
Example images in Clutter Complexity experiment. Images with the running man sculpture as presented in the following seven experimental conditions: from left to right, high-resolution control without background clutter and low-resolution images with 0%, 5%, 10%, 15%, 20%, and 25% background clutter complexity.

Testing was conducted online by crowdsourcing using Amazon Mechanical Turk (MTurk)^26,27^ to effectively reach a high number of participants. To prevent any learning effect with repeatedly seen objects, we ensured that each subject would only see each object once. Static images taken from the central viewpoint (Fig. 1) were used. For each object, the following seven experimental conditions were tested: six conditions with 20 × 20-resolution simulations of the object in front of six levels of background complexity (0%, 5%, 10%, 15%, 20%, and 25%), and a control condition with the high-resolution images (480 × 480) without background clutter (Fig. 3). To ensure each individual subject saw each object only once, the 35 × 7 = 245 images were split into seven sets, and each set contained 35 images, one for each of the 35 objects taken at only one of the seven experimental conditions. Each of the seven sets was presented to 10 subjects to acquire an average recognition rate for each object × background combination. A total of 70 subjects were required.

The Unique Turker service (http://uniqueturker.myleott.com/) was used to limit each subject to one set and each set of images was published separately. Because the presented images were intended to subtend approximately 20° diagonally, subjects were guided to calibrate the display image size by matching an oval-shape displayed on the screen with their closed fist at arm's length and asked to maintain their sitting distance from the monitor over the course of the task. Four practice trials with objects different from the 35 testing objects were followed by the 35 experimental trials in random order across subjects (1 trial for each object in 1 of 7 conditions). Each image was presented for up to 10 seconds, allowing sufficient time for viewing, and then subjects responded by typing the name of the object without a time limit and pressed “Enter” to move to the next trial. No feedback was given following the trial. If words of less than three letters were submitted, the response was rejected, and the subject was asked to re-enter the object name. Subjects were encouraged to guess if they did not recognize the object. Each subject was compensated $1.50 if they completed all 35 trials and provided reasonable responses, especially in the high-resolution control trials where objects should have been more easily recognizable (see the first image in Fig. 3).

Three experimenters, who tried to hold consistent criteria across the conditions and subjects, scored subjects' typed responses independently. When scoring responses, experimenters used their best judgment about whether a response was correct, and also considered the way the objects might have looked to viewers in the images. If a response was identical with our name for the object, it was marked correct. If a response was mostly correct but included some additional details (e.g., “man in hat” for the “hat”) it was scored as correct because the hat sat on a mannequin head in the image. Subjective judgment was necessary if an object looked more ambiguous in an image (e.g., the football was round/oval shaped with little to no texture detail in the low-resolution image so common responses were “lemon” or “egg”) in which case the scorers had to make a decision based on how closely the image resembled the response. To account for disagreement between scorers, a response was marked correct if it was scored as such by at least two of the three scorers. The recognition rate for each object under each condition was calculated as the number of subjects who successfully recognized the object divided by 10, the number of subjects who were tested with each object and condition. The effects of background complexity levels on recognition rates were analyzed using a nonparametric method, the Wilcoxon signed-rank test,^28^ given the nonnormal data distribution. The symmetry assumption of the Wilcoxon signed-rank test was evaluated using the MGG test (a test of symmetry about an unknown median).^29^ The Bonferroni correction was used to correct for multiple comparisons.

### Recognition Rate

**Figure 4:**
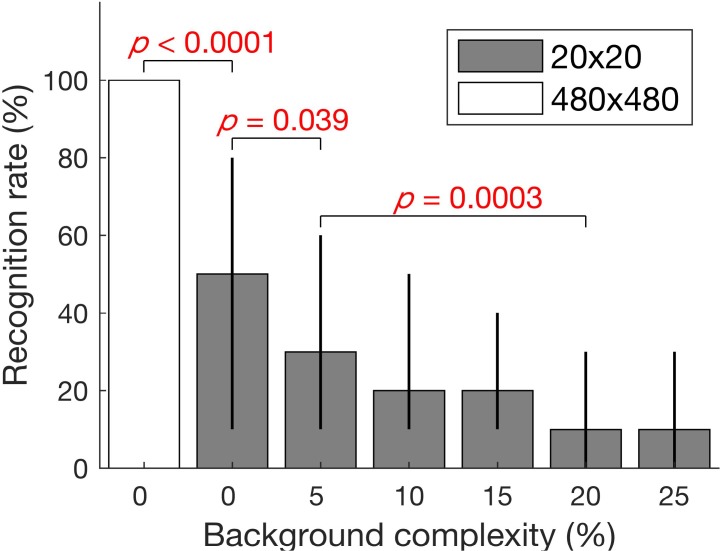
Median recognition rates with different resolutions and background clutter complexities. Background clutter reduces the recognition rate in low-resolution images, though the effect was smaller than the effect from the resolution change (from 480 × 480 to 20 × 20 both without background clutter). The *P* values were calculated using the Wilcoxon signed-rank test with paired samples and were Bonferroni corrected for multiple comparisons. *Error bars* represent the interquartile range.

All 70 MTurk subjects received the full compensation. The median recognition rate across the 35 control images (the recognition rate for each image was calculated across the subjects) at high resolution (480 × 480) without background clutter was 100%. The decrease in resolution to 20 × 20, without background clutter, reduced the median recognition rate in half to 50% (Fig. 4). Comparing the recognition rates of the high-resolution images with those of the low-resolution images, the *P* value of a paired, one-sided Wilcoxon signed-rank test was less than 0.0001 (*z* = 4.83, the MGG test for symmetric differences *P* = 0.69). Among the conditions at the low resolution (20 × 20), when comparing with the no-clutter condition, the 5% background complexity reduced the median recognition rate from 50% to 30% (*z* = 2.42, Bonferroni corrected *P* = 0.039, symmetry test *P* = 0.37). When the background complexity was further increased to 20%, the median recognition rate reduced from 30% to 10% (*z* = 3.83, Bonferroni corrected *P* = 0.0003, symmetry test *P* = 0.16).

The Clutter Complexity experiment was used to select one level of background complexity for each object to be used in the Motion Parallax experiment (the recognition rates for each object are shown in Supplementary Appendix Fig. A1). To allow room for improvement, the complexity level that led to the lowest recognition rate was selected for most of the objects for the next experiment. For those having comparable recognition rates at multiple background complexity levels, one complexity among the multiple levels was randomly selected. The selected background complexity level for each object is indicated with a filled black circle in Supplementary Appendix Figure A1.

## Motion Parallax Experiment

### Impact of Motion Parallax on Recognition

The Motion Parallax experiment was designed to test whether the motion parallax with simulated OI stabilization would improve object recognition in a cluttered environment.

### Apparatus

The Oculus Rift (Oculus VR, Irvine, CA) head-mounted display (HMD) was used to track subjects' lateral head positions and to display the corresponding precaptured images. The virtual world displayed in the Oculus Rift was set to simulate the physical environment where the image set was captured. The images subtended 24° of visual field in the Oculus Rift to match default FoV in the BrainPort. The image area surrounding the displayed images was shown in black. The same images were displayed to both eyes, and thus there was no depth information. This also prevented a potential rivalry between the test image and the blank dark screen if only one eye was presented with the image. The measured lateral head movement ranged ±12 cm from the central position corresponding to the middle viewpoint, and every 3-cm interval for the head positions corresponded to one viewpoint. Hysteresis control was applied to reduce the effect of tracking noise. The head rotations were not tracked, and only the lateral positions affected the change in image presentation. Images were presented in a small FoV, and no obvious benefit could be achieved through eye scanning. Therefore, subjects completed the task without enforced fixation, and eye movements were not tracked.

### Experimental Design

The same 35 objects used for the Clutter Complexity experiment were used. Two factors were included in the experimental design, (1) the background clutter (with or without), and (2) the head scanning conditions (static, coherent, or random scanning). For the conditions without background clutter, the images displayed were taken in front of a uniform gray background; for the conditions with background clutter, one background complexity with low recognition rates selected in the Clutter Complexity experiment was used.

Three head scanning conditions were tested—static from a single viewpoint, coherent nine viewpoints corresponding to subjects' lateral head positions, and nine viewpoints randomly displayed. The images displayed for the static condition were from the central viewpoint regardless of subjects' head movements. The condition with coherent motion parallax cues was displayed from the pre-captured nine-viewpoint images corresponding to subjects' head positions. Therefore, subjects would perceive viewpoint shifts coherent with their head movements. In the random scanning condition, the image presented at each head position was selected randomly from the nine-viewpoint images: the association between the image viewpoint index and the head position were preset through random sampling without replacement. The associations were thus constant across subjects but not objects. The overall parallax effect of the OI being stabilized and the background moving was maintained in the random scanning condition, but neither the background image movement nor the multiple viewpoints of the OI were coherent with the head movements. A total of six experimental conditions (2 backgrounds × 3 head scanning conditions) were presented as shown in Supplementary Movie S1, QuickTime movie file.

### Procedure

Sixty normally sighted subjects (12 males, average age 27), recruited from the Schepens Eye Research Institute and the New England College of Optometry completed the object recognition task (10 subjects per condition for each object). The subjects were first screened to ensure visual acuity of 20/30 or better with habitual correction (30 subjects were wearing contact lenses) and a visual field wider than 50° FoV. Subjects passed the Mini-Mental State Examination (MMSE). Subjects were seated, fitted with the HMD, and underwent calibration to ensure the HMD was within the field of the Oculus lateral movement sensor. The experimenter demonstrated the lateral head movement that would induce the motion parallax and encouraged subjects to use this motion when viewing images.

The first six trials were practice trials, one for each experimental condition, and were followed by 35 test trials, one for each test object. The objects presented in practice trials were different from the 35 test objects. Each subject only saw each test object once. Each object under each experimental condition was seen by 10 subjects (thus 60 subjects were required for the 6 experimental conditions). The conditions were evenly and randomly distributed for all subjects—similar number of trials for each experimental condition. The order of object presentation was random across subjects.

A blank screen with the trial identification number was displayed at the beginning of each trial, and the subjects were instructed to initiate the image presentation when they were ready (by pressing a button on the Oculus handheld remote). They could take as long as they needed to inspect the images with their lateral head movements. The displayed image sequence and the corresponding time interval for each image were logged to monitor the subjects' head movements when exploring the images. Once they decided on the name of the object, they pressed the Oculus remote button again to end the image presentation, which recorded the response time for the trial. The subjects then verbally reported the name, which was recorded by the experimenter. The subjects were encouraged to guess and only to provide one answer that they thought was the most plausible. No feedback was given.

### Analysis

The recorded naming responses were independently scored by three experimenters, as done for the Clutter Complexity experiment, and responses scored as correct by at least 2 out of 3 experimenters were coded as correct. An average recognition rate for each object under each condition was the number of subjects who successfully recognized the object divided by 10. The recognition rates among the six experimental conditions were analyzed using the Wilcoxon signed-rank test and the Bonferroni correction for multiple comparisons was applied. For the response time, a two-way repeated measures ANOVA (background conditions and scanning conditions as the within-subject factors) was conducted. The post hoc contrasts were Bonferroni corrected for multiple comparisons. Fourier analysis was conducted on the displayed image sequence (as quantized head movement data) to analyze the head movement patterns. The Wilcoxon signed-rank test was used to compare the head movement pattern among the experimental conditions.

### Recognition Rate and Response Time

For individual subjects, the average recognition rate across the objects was 33%. Of the 35 objects, the median number of recognized objects was 11.5 (range, 3–22). We calculated a bounded range of valid recognition rates by adding 1.5 times the interquartile range (IQR = 5.5) to the third quartile (14) for the range maximum and subtracting the same modifier from the first quartile (8.5) for the range minimum. Because no subject had a recognition rate outside these bounds (0.25–22.25), no subject was excluded as an outlier in the analyses.

**Figure 5:**
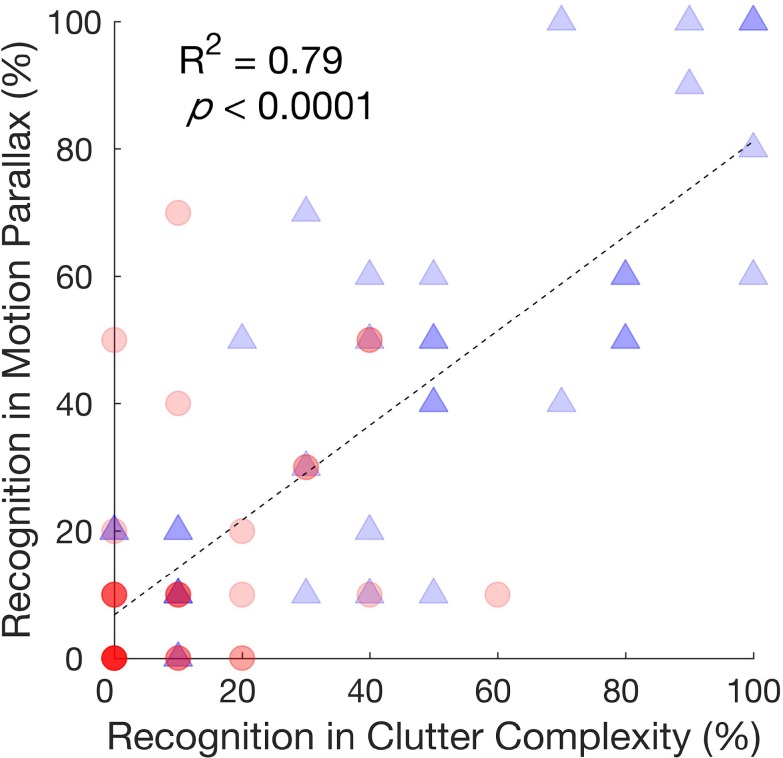
Recognition rate of the same images collected in the Clutter Complexity (x-axis) and the static condition of the Motion Parallax (y-axis) experiments. The *blue triangles* are from the no clutter and static condition, and the *red circles* show the recognition rates with the cluttered background. Each point represents the responses to one object under the corresponding condition (70 points in total). The points may overlap indicating the same recognition rates among the objects. The darker the icon is, the more points are overlapping. For example, the majority recognition rates from the cluttered condition are either 0% or 10%. The recognition rates in the two experiments are significantly correlated.

Next, we verified that when provided with the same 20 × 20 images, responses collected in the Clutter Complexity and the Motion Parallax experiments were correlated (Fig. 5), despite the different interfaces and subjects (MTurk crowdsourcing and HMD, respectively). Specifically, the recognition rates of conditions with no clutter and the selected level of complexity from the Clutter Complexity experiment were compared with those in the static conditions from the Motion Parallax experiment. When there was no clutter (blue triangles in Fig. 5), the coefficient of determination (*R*^2^) between the recognition rates in the two experiments was 0.80 (*P* < 0.0001). With clutter (red circles in Fig. 5), over 80% of the objects have a recognition rate smaller than 20% (either 10% or 0); the *R*^2^ here was weaker (0.35), but the relationship was still significant (*P* = 0.040).

**Figure 6:**
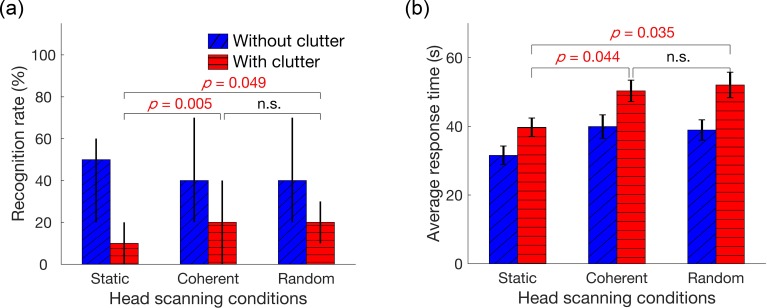
Median recognition rate and average response time for each condition. (a) Median recognition rates across the 35 objects for the six experimental conditions. Background clutter significantly reduces the recognition rates. Motion parallax (both coherent and random scanning) significantly improved the object recognition rate. *Error bars* represent the interquartile range. (b) Average response times for the six conditions. The conditions with clutter show a significantly longer response time than the conditions without clutter. Subjects also tended to spend more time in the conditions with multiple views when compared with the static conditions. The *P* values were Bonferroni corrected. *Error bars* are standard error of the mean (SEM).

Figure 6a shows median recognition rates across the 35 objects for each experimental condition (results for the individual objects can be found in Supplementary Appendix Fig. A2). Because the normality assumption was violated by the recognition rate data, the Wilcoxon signed-rank test was used for statistical analysis. The recognition rate was lowest for the condition with static cluttered images (median 10%), while all conditions without background clutter showed a median recognition rate of 40%. Background clutter significantly reduced the recognition rates, among the static conditions (symmetry test *P* = 0.88), the approximate value of the *z*-statistic was 4.64 with Bonferroni corrected *P* < 0.0001; for the coherent head scanning conditions (symmetry test *P* = 0.97), the *z*-statistic was 3.64 with corrected *P* = 0.0012; the *z*-statistic was 3.71 with corrected *P* = 0.0001 for the random head scanning conditions (symmetry test *P* = 0.39). With background clutter, both head scanning conditions showed improved recognition rates in comparison with the static condition (median recognition rate 10%): coherent scanning improved the median recognition rate to 20% (*z* = 3.28, corrected *P* = 0.005, symmetry test *P* = 0.47); and with the random scanning, the median recognition rate increased to 20% (*z* = 2.55, corrected *P* = 0.049, symmetry test *P* = 0.90). No significant difference in performance was found between the clutter coherent (median recognition rate 20%) and random viewing (median 20%) conditions (*z* = 0.44, *P* > 0.05, symmetry test *P* = 0.45). Without background clutter, the recognition rates were not significantly different among the three head scanning conditions, the median recognition rates were 50%, 40%, and 40% for the static, coherent, and random scanning conditions, respectively (*P* > 0.5 for all pairwise comparisons). An alternative analysis calculating the recognition rate for each subject across the objects, and thus treating the repeated measures over subjects is provided in Supplementary Appendix – Alternative analysis of recognition rates, which led to the same conclusion.

The response times across the 10 subjects for each object and experimental condition were averaged for statistical analyses (Fig. 6b). On average, 42 seconds were needed for each trial; subjects spent time to move laterally back and forth to view images from various viewpoints as instructed. A two-way repeated measures ANOVA (2 backgrounds × 3 head scanning conditions as the within-subject factors) was conducted for the response times. The data were approximately normally distributed in the groups (Lilliefors test) and satisfied the sphericity assumption based on the Mauchly's test (for the factor of scanning condition, test statistic *W* = 0.98, *P* = 0.69; for the interaction term, *W* = 0.98, *P* = 0.71). Both background conditions (*F*(1, 34) = 10.23, *P* = 0.003) and scanning conditions (*F*(2, 68) = 9.82, *P* = 0.0002) significantly influenced the response times. No interaction was found between the two factors (*F*(2, 68) = 0.54, *P* = 0.59). All conditions with background clutter showed significantly longer response times (on average, 47 seconds) than the conditions without clutter (∼37 seconds, *t*(104) = 4.28, corrected *P* = 0.0004). Subjects spent more time in the conditions with multiple views when compared with the static conditions (36 seconds): the coherent multiple-view conditions were on average 9.5 seconds longer and the random scanning conditions were approximately 10 seconds longer. The response time of the no-clutter static viewing condition was the shortest (32 seconds). With background clutter, the post hoc contrasts showed that significantly longer times were spent for the coherent scanning (*t*(34) = 3.01, corrected *P* = 0.044) and the random scanning conditions (*t*(34) = 3.10, corrected *P* = 0.035) when compared with the static condition.

### Head Movements Analyses

The displayed image sequence for each trial was triggered by subjects' lateral head movement, and thus indicated quantized head positions. For each trial, the head movement range was first characterized using the number of displayed unique images from the preset nine viewpoints. The wider the subject's head shifted laterally, the more unique viewpoint images were displayed. On average, approximately five of nine frames were explored covering approximately 12-cm lateral head shift. Slightly but significantly more frames were explored in the conditions with clutter, and more frames were seen under coherent or random scanning than the static conditions (see Supplementary Appendix Fig. A3 for more details). The median main scanning frequency was approximately 6 cycles/min, which means that approximately 10 seconds was spent on average by subjects to complete one cycle (Supplementary Appendix – Results of head movement analysis).

## Discussion

### Visual Prostheses Evaluation

We tested a newly proposed strategy of OI-stabilized motion parallax^13,21^ to declutter backgrounds and aid in object recognition in low-resolution images. This strategy may be applied to future visual prostheses. A visual prosthesis should provide spatial and temporal characteristics similar to those of the human vision system so that it is able to assist in visual tasks, such as learning, adapting, and generalizing from trained objects to untrained or even unfamiliar objects.^30^ Bearing such goals in mind, we developed a testing environment that avoided repeatedly using trained objects in the testing. Object recognition refers to a connection between a previously encountered stimulus and a new encounter with the same/similar stimulus.^31^ Prior studies with visual prostheses (e.g., Alpha IMS, Argus II) and SSDs (BrainPort) reported investigating object recognition, but used a few pretrained objects in their performance evaluation.^3,11,12,32^ In the literature, this is referred to as pattern or object discrimination^33^ in distinction from object recognition.

Because we are interested in object recognition performance with and without decluttering provided by motion parallax, we designed the experiments to ensure a recognition task and not a mere discrimination task. Our subjects saw each new (not previously presented) object only once across all trials. This methodology has been useful for our study using degraded images to simulate prosthetic vision with normally sighted subjects. However, it requires a large number of subjects, which will be impractical in clinical trials with implanted subjects or even with blind users of SSDs. Alternately, one can use a limited number of subjects if a large supply of images is available for repeated testing. In preparing this study and more recent work, we realized that selecting and imaging a large number of objects for such testing may not be simple for either real or virtual objects. A proper and efficient methodology for confirming the visual system characteristics, that is, the abilities of a functional vision system of visual prostheses, is needed for future evaluations.^30^

### Background Clutter Disrupts Recognition in Simulated Prosthetic Vision

**Figure 7:**
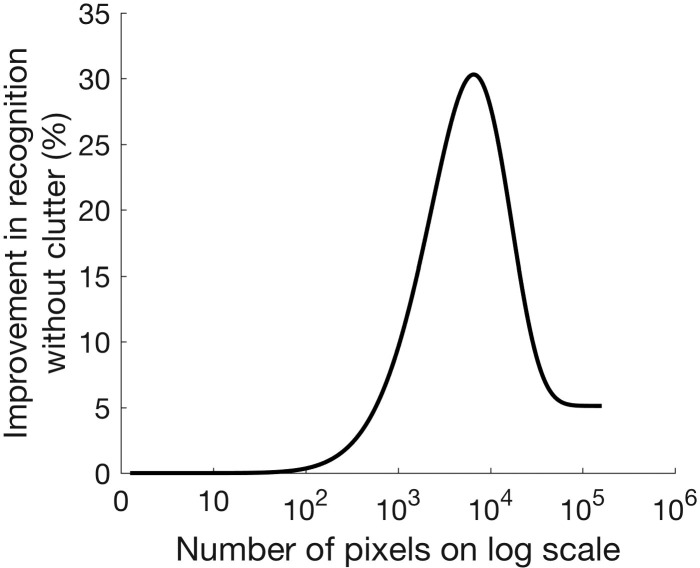
The difference in object recognition between with and without background clutter (*Recog_noclutter_* − *Recog_clutter_*) for binary edge images as a function of resolution, calculated from the data presented in figure 8 of Jung et al.^10^

Background clutter hinders subjects' ability to recognize objects,^9^ and visual prostheses are more susceptible to this problem due to the low resolution and dynamic range of current devices.^10^ These limitations have been shown with simulated prosthetic vision where subjects have significantly worse recognition performance with background clutter compared with no clutter in a static scene. Jung et al.^10^ investigated the effect of resolution on recognition improvement in binary edge images without and with clutter (Fig. 7). Their results show that given a fixed FoV of 10° × 7°, the difference in recognition rates between without and with clutter first increases and then declines as the resolution increases. The improved recognition due to decluttering reaches maximum around a resolution of 10,000 pixels. The benefit of decluttering starts to decline for higher resolution, as the difference saturates at a resolution of 100,000 pixels or higher.

Results from both our experiments showed that background clutter yielded lower recognition rates compared with those with clutter-free backgrounds in the static condition, further emphasizing that background clutter diminishes the recognition performance. In the Clutter Complexity experiment, from the 20% background complexity (median recognition rate 30%) to no clutter (median recognition rate 50%), recognition rates differed by 20%. In the static condition of the Motion Parallax experiment, recognition rates with background clutter (median 10%) improved to 40% without background clutter, a 30% difference. The difference in the latter was larger because the background complexity with the lowest recognition rate was selected for the experiment. Note that the improvement in recognition rate for the 400-pixel images we used is expected to be only 5% for binary images as shown in Figure 7 based on Jung et al.^10^ Although the magnitude of object recognition rate may vary with experimenter scoring and subjects in different studies,^34^ this large difference may be accounted for by the dynamic range difference. Here, we used a much higher dynamic range (256 levels) compared with the binary images used by Jung et al.^10^ Resolution and dynamic range can be traded-off, as in dithering or halftone,^35,36^ and thus our results could be comparable to those of higher resolution binary images described in Figure 7. The level of improvement we found with no cluttering is close to the level of improvement with much higher resolution in binary edge images (∼10^3.5^–10^4^ in Fig. 7).

### Decluttering Improves Recognition of Fixated Object With Background

Various techniques have been proposed to overcome the effect of background clutter.^10,14,37–41^ Our proposed motion parallax method takes advantage of the visual system's ability to declutter based on differential movements of object and background. In the Motion Parallax experiment, we used the Oculus HMD to simulate motion parallax with fixated objects using subjects' head movements. With background clutter, the object recognition performance improved from 10% with static viewing to 20% with coherent multiple viewpoints. The simulated motion parallax with OI-stabilization did allow for better performance by presumably providing subjects with cues to aid in separating object from background. This is not very surprising as we used the visual systems of normally sighted subjects to perform the interpretation. This is encouraging but it does not assure that a similar improvement may be achieved with a retinal prosthesis or stimulation of the tongue (e.g., the BrainPort). Further testing using a similar paradigm is required.

Although motion parallax decluttering almost doubled the recognition performance (from 10%–20%), it did not achieve the same level of performance as the conditions without clutter (40%). This suggests that even with these better pixelated images and normal visual processing, the decluttering effect of motion parallax is partial and better performance may be achieved if the clutter is further eliminated or reduced. Does this mean that methods to remove background clutter, such as in Jung et al.^10^ and Dagnelie et al. (*IOVS*. 2016;57:ARVO E-Abstract 5167), are preferred or should these be combined with motion parallax? Further research is required to provide an answer to this question. Meanwhile, the use of head movements combined with our proposed OI-stabilization provides additional benefits to users, such as multiple viewpoints and depth cues, which should not be overlooked.

For example, the viewpoint change from head scanning may support better recognition due to the availability of multiple views of an object providing additional information. Bambach et al.^42^ showed that the improvement in recognition due to multiple viewpoints is higher when the relationship between the observer and the object is consistent. Such a consistent relationship occurs when the observer is moving himself/herself, as in motion parallax, or the observer manually manipulates the object on his/her own. The benefit of the multiple viewpoints declines if they are generated by independent movement of the object.^43^ However, our results with the very low-resolution images failed to show the performance improvement due to multiple viewpoints: among the condition without clutter, the recognition rates were not improved through scanning with multiple viewpoints (all medians ∼40%–50%). This may be due to the coarse image pixels, limiting the changes of neighboring viewpoints and making them rather hard to interpret.

We also found that the random scanning condition improved recognition performance almost as well as the coherent condition. The lack of difference here may suggest that a matching between the user's self-motion and the acquired images was not important to recognize the OI. However, the coherent scanning may serve other important functions. Besides avoiding the potential risk of motion sickness, the correspondence between the self-motion and the acquired sensory information yields crucial cues in distinguishing the self-motion from the external object motion (e.g., OI-induced motion). The external object motion was also found to be beneficial in recognizing low-resolution blurry images.^44^ Further studies are necessary before we conclude prematurely that coherence of motion is of no value.

While motion parallax may aid decluttering and improve object recognition, it inherently takes time for the subjects to move their heads and create the movement parallax. In the Motion Parallax experiment, subjects took approximately 10 seconds longer to respond in background clutter conditions than in no-clutter conditions. Subjects on average spent approximately 10 seconds to complete one cycle of lateral head movements. The longer response times may reduce the effectiveness of motion parallax decluttering for object recognition.

### Summary

We demonstrated the usefulness of OI-stabilized motion parallax for improving object recognition, presumably by supporting object and background separation. The improvement was demonstrated with a normal visual system and needs to be repeated with true visual prostheses or SSDs. Even with normal sight the improvement was modest, leaving room for further improvement with additional approaches for background clutter removal.

## Supplementary Material

Supplement 1

Supplement 2
