# Supplementary material for: Motion Parallax Improves Object Recognition in the Presence of Clutter in Simulated Prosthetic Vision
Source: Transl Vis Sci Technol. 2018 Oct 29;7(5):29. doi: 10.1167/tvst.7.5.29 (PMC6205682; doi:10.1167/tvst.7.5.29)
Supplement: Supplement 2 [file tvst-07-05-19_s02.docx]

# Appendix

## Object images captured with BrainPort V200 camera

Full list of objects captured with the BrainPort V200 and the distances at which the objects were placed from the camera based on object size. Object order matches the order used in Fig. A1.

| Object | Distance (cm)  from camera to object | Object | Distance (cm)  from camera to object |
| --- | --- | --- | --- |
| mason jar | 50 | lemonade bottle | 70 |
| teapot | 50 | rubber duck | 30 |
| rat | 50 | cow | 30 |
| wooden body | 70 | truck | 30 |
| mug | 50 | boot | 70 |
| scissors | 30 | turtle | 30 |
| headphone | 70 | tree | 50 |
| plant | 70 | tea tin | 50 |
| full wine glass | 70 | spray bottle | 70 |
| teddy bear | 70 | pear | 30 |
| glue bottle | 50 | hat | 70 |
| fish | 70 | lamp | 70 |
| pot | 70 | stapler | 50 |
| purse | 50 | mannequin head | 70 |
| sun glasses | 70 | tape dispenser | 50 |
| sneaker | 70 | football | 50 |
| flash light | 70 | coffee cup | 50 |
| building | 30 |  |  |

Images of the 35 objects captured for both experiments in front of various background complexities are shown below. The leftmost image is the high-resolution control (480×480). The second image is low resolution (20×20), no background, and subsequent images are low resolution with 5%, 10%, 15%, 20%, and 25% background complexities, respectively. Object order is arranged to match that used in Fig. A1. Different backgrounds were used across the objects although for the same complexity level.


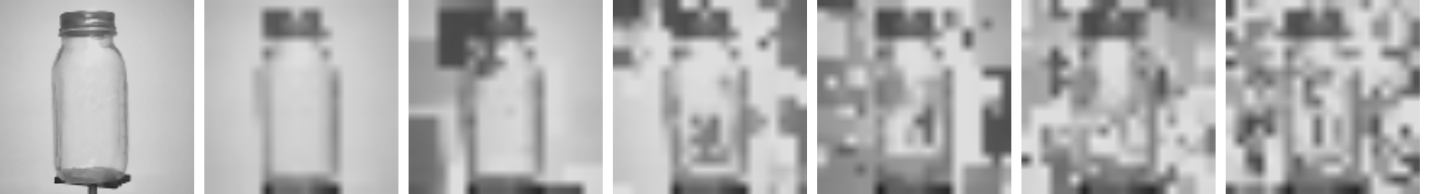


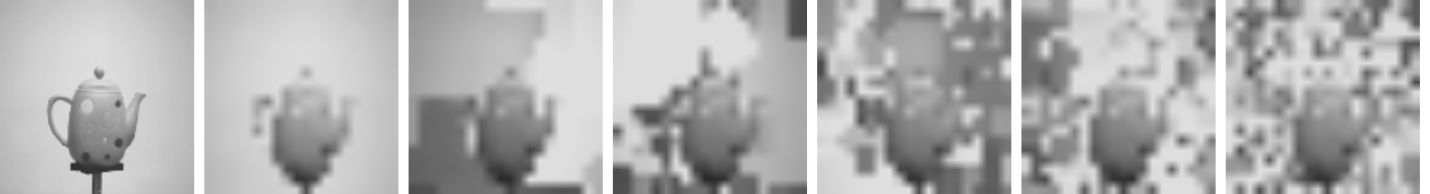

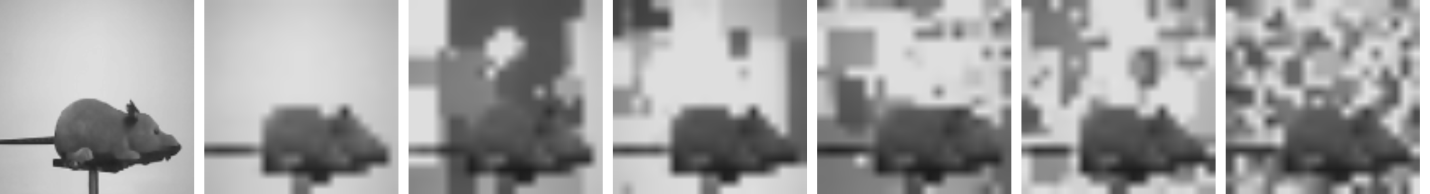

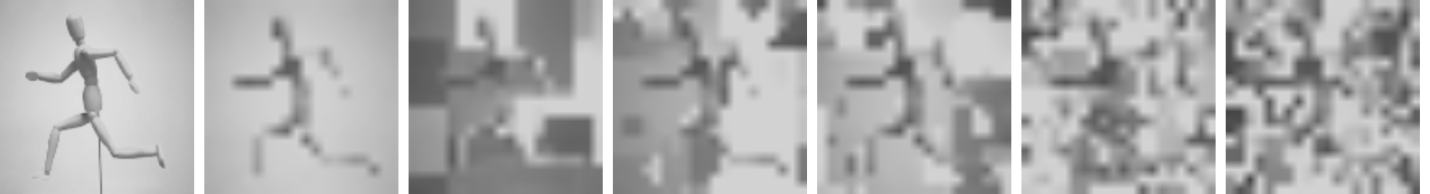

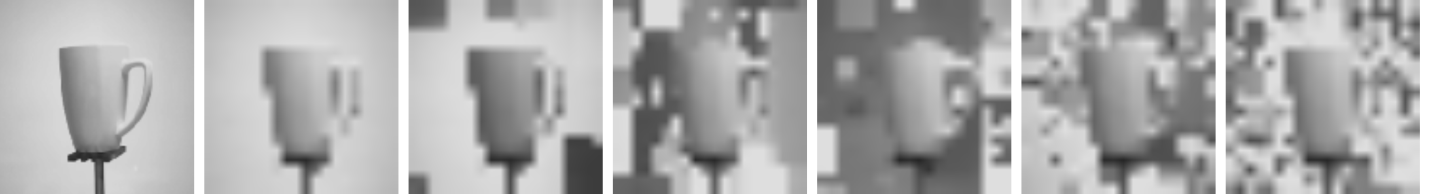

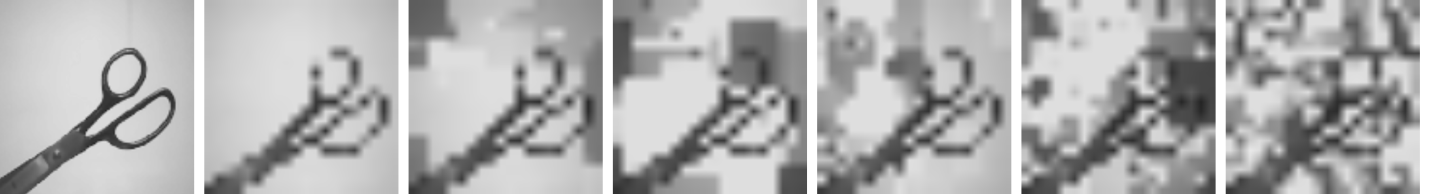

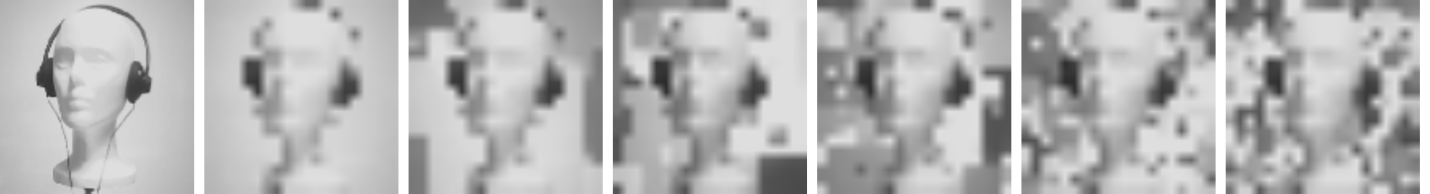

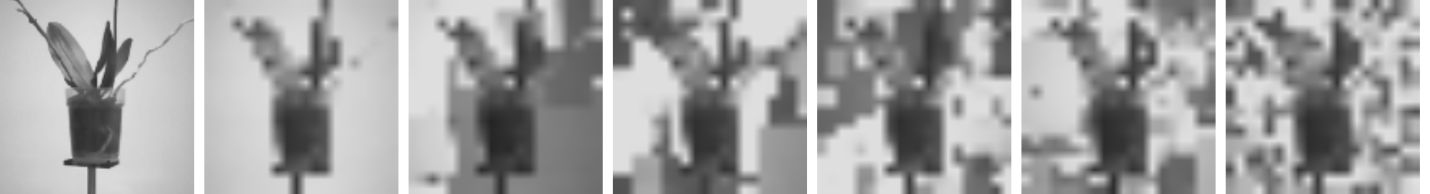

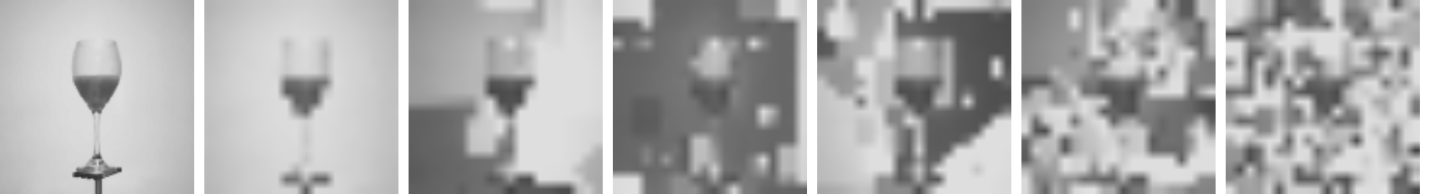

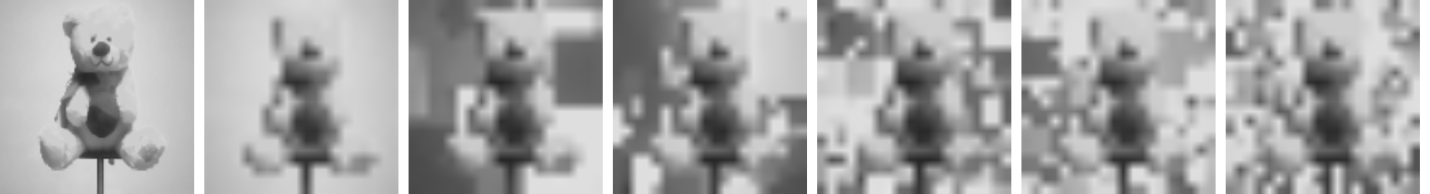

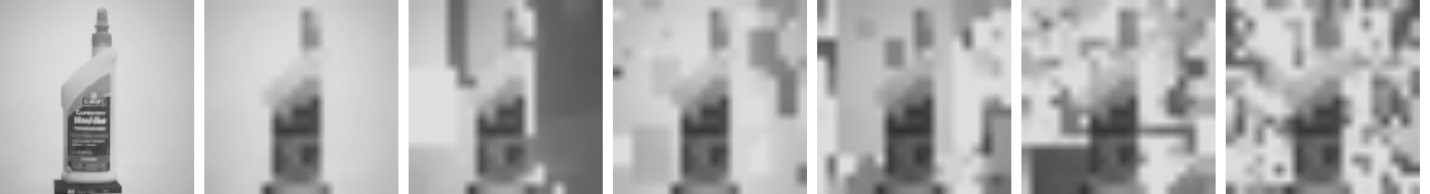

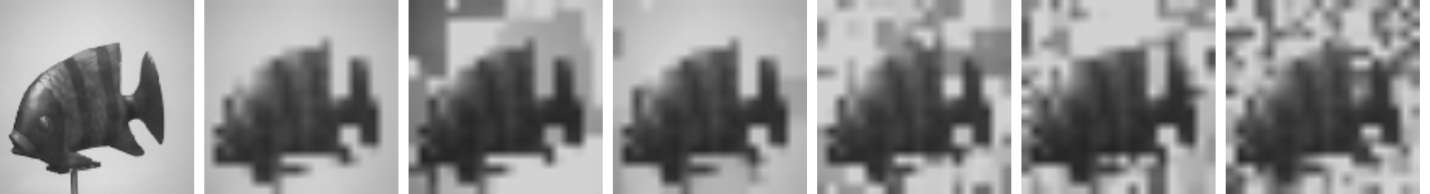

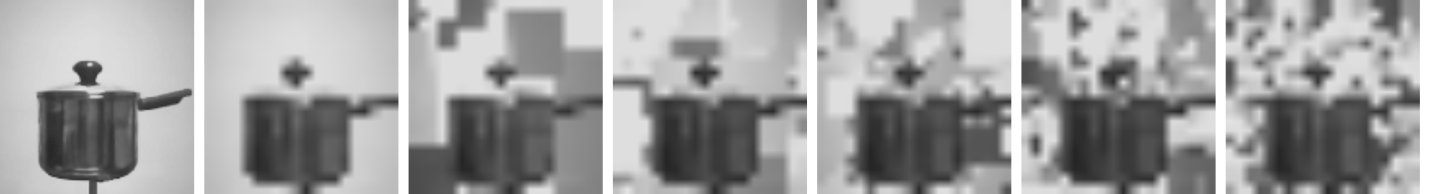

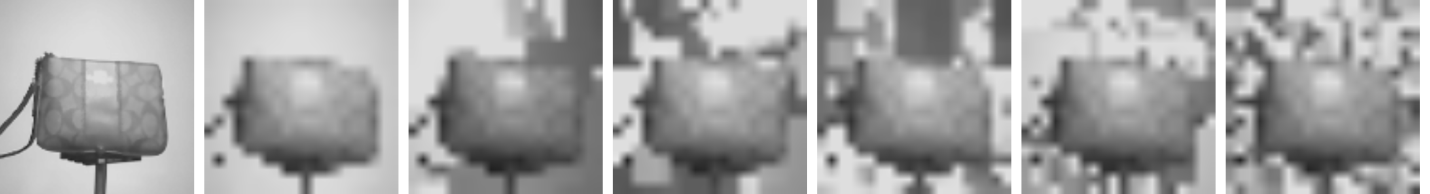

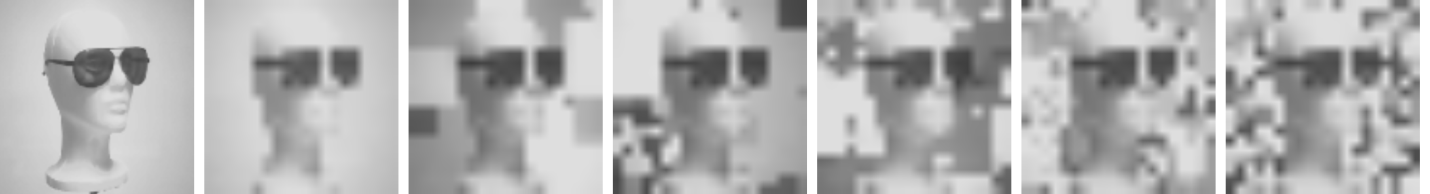

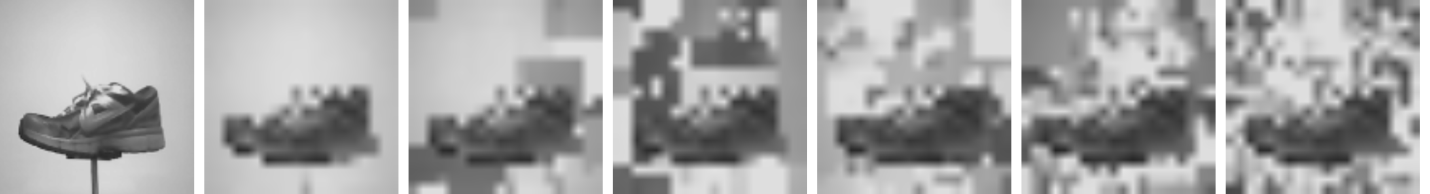

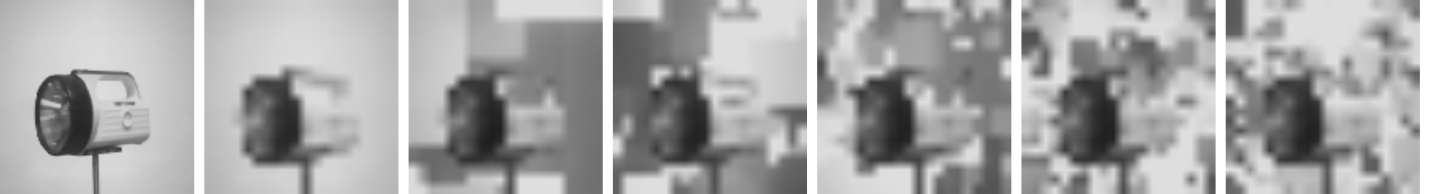

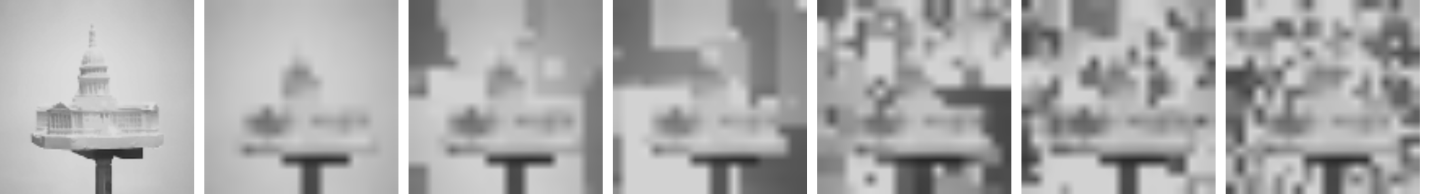

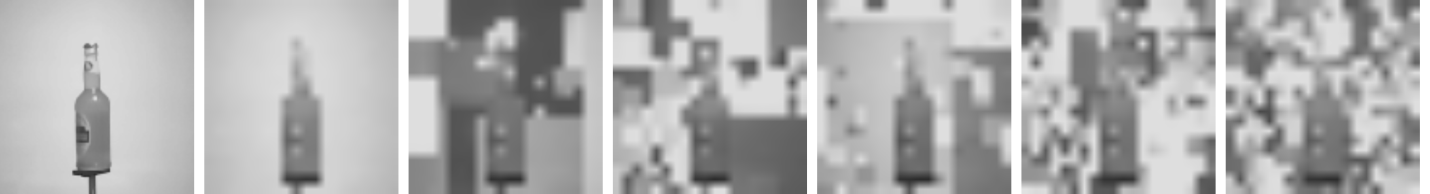

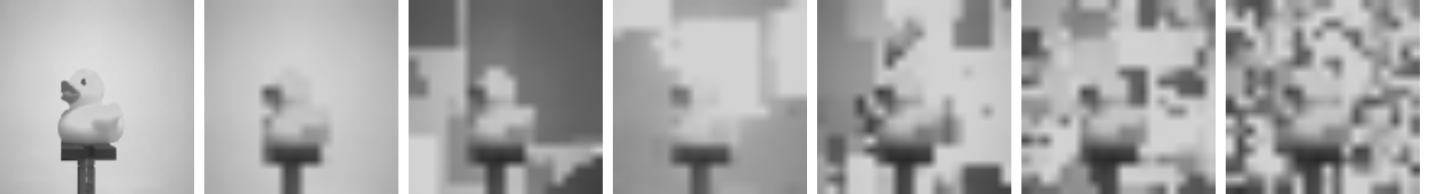

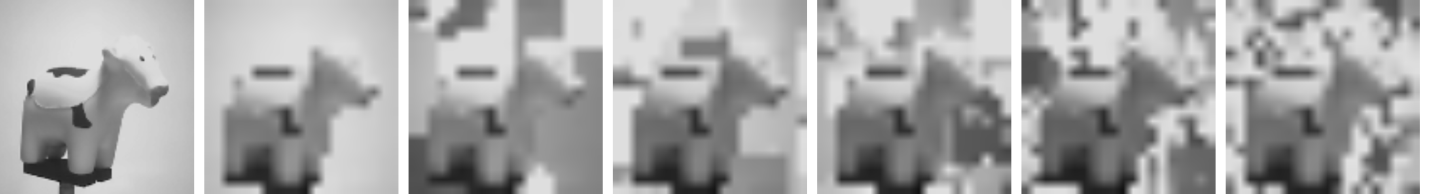

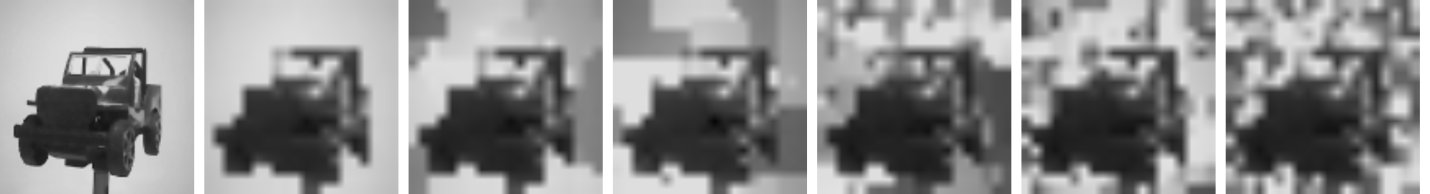

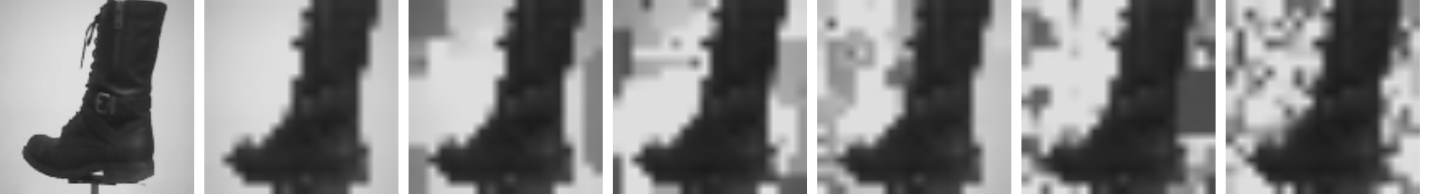

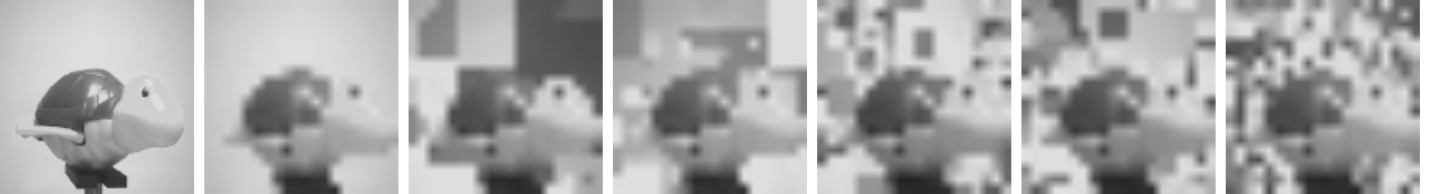

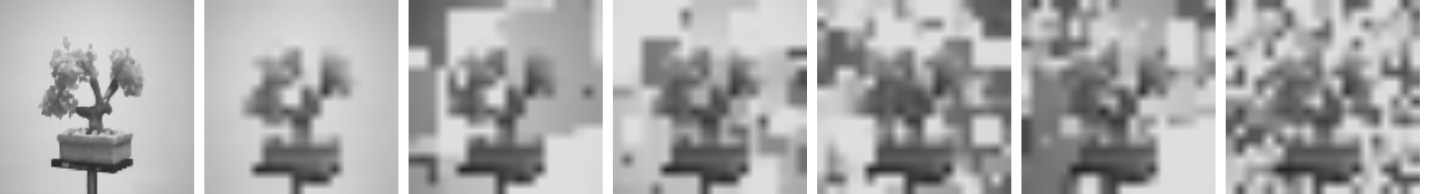

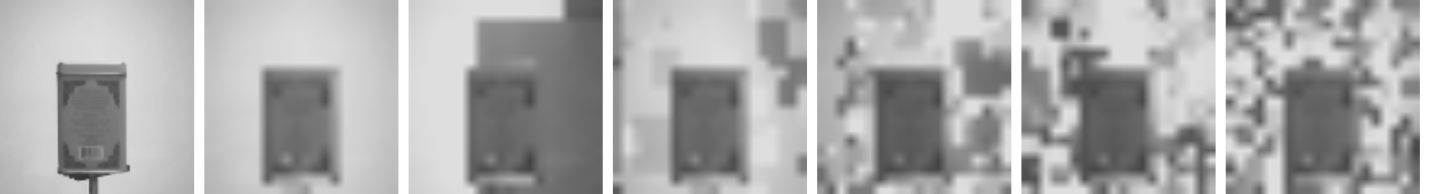

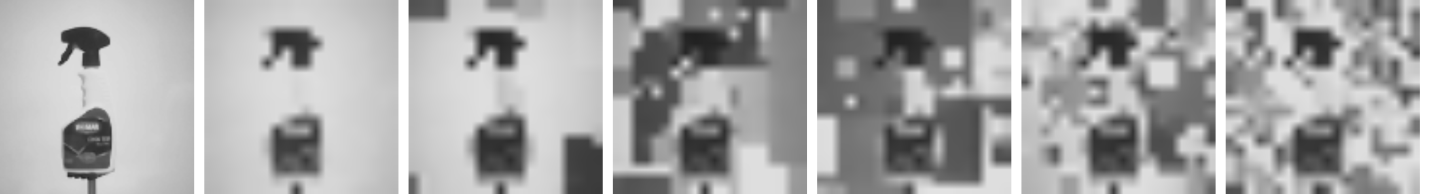

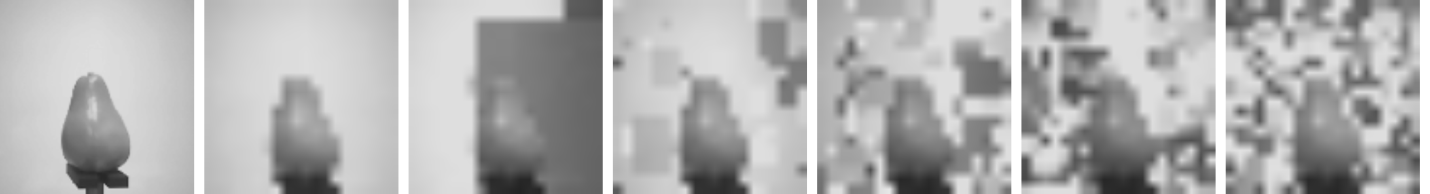

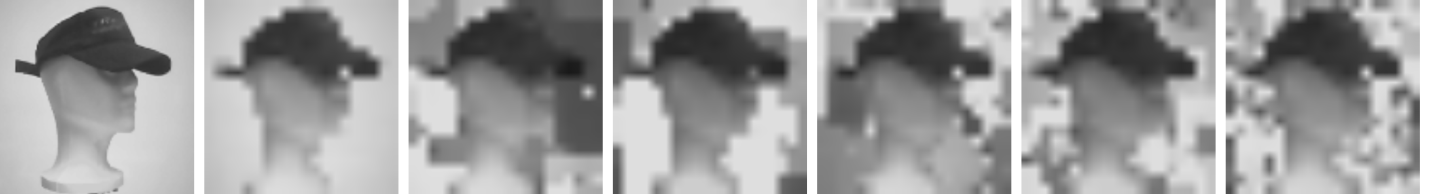

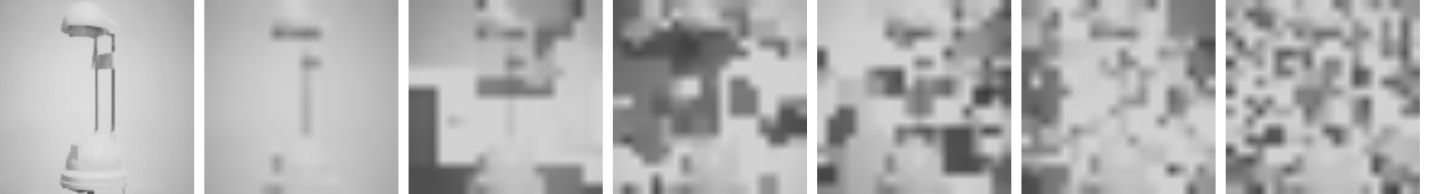

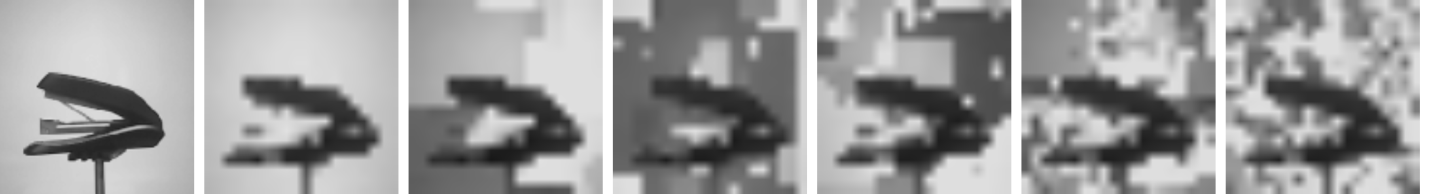

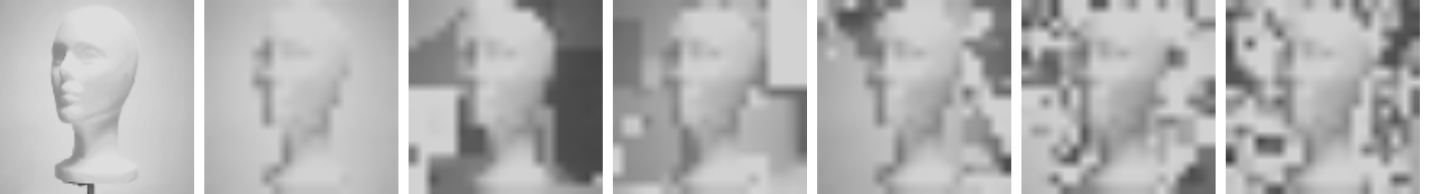

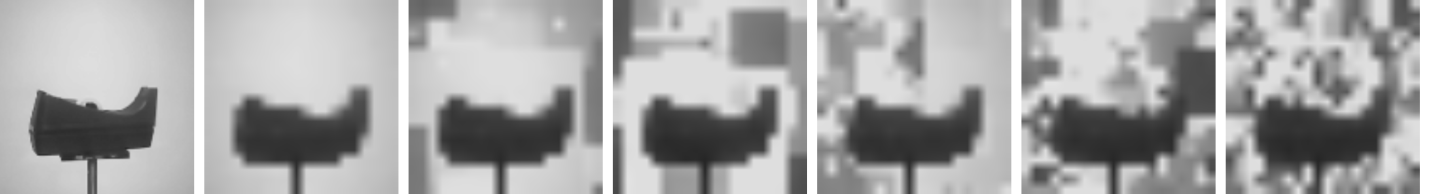

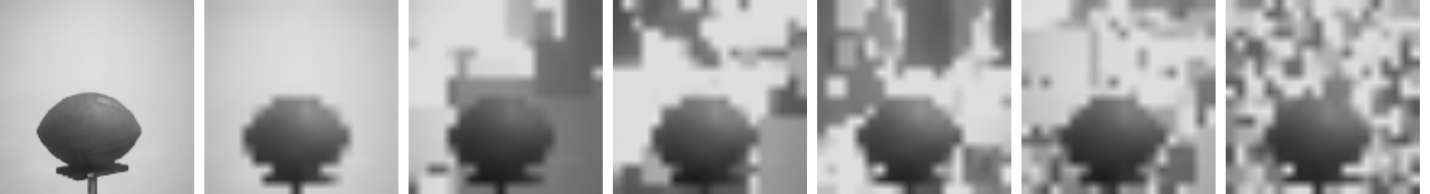

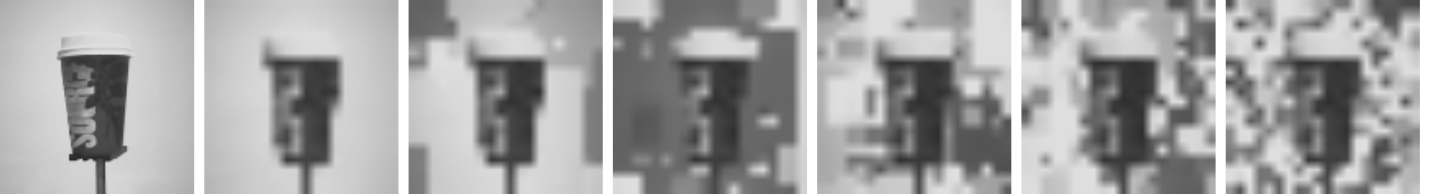


## Recognition results of individual objects

Figure A1 shows the recognition rates of each individual object and condition. For some objects, recognition rates in low resolution were low across all background complexity conditions, indicating the resolution was a major factor hindering recognition. These included the tape dispenser, pear, lamp, building, turtle, and flashlight. Objects such as the mannequin head, stapler, rubber duck, boot, and coffee cup showed a non-monotonic effect of the background complexity, among which, the rubber duck and coffee cup had better recognition with one certain background, perhaps due to higher contrast provided at that background image. To quantify the effect of background complexity, the recognition rates as a function of the background complexity levels were fitted to a logistic model for each object (dashed lines in Fig. A1). A logistic regression across the 35 objects also showed that the odds of the object being recognized in 5% background complexity was 0.55 times the odds of being recognized in no-clutter ($\hat{\beta}=-0.60, z=-3.83, p=0.0001$); that is, the odds of being recognized was 45% lower for 5% background complexity than no-clutter. The odds of being recognized was 72% lower for 25% background complexity than no-clutter ($\hat{\beta}=-1.27, z=-7.47, p<0.00001$).

| 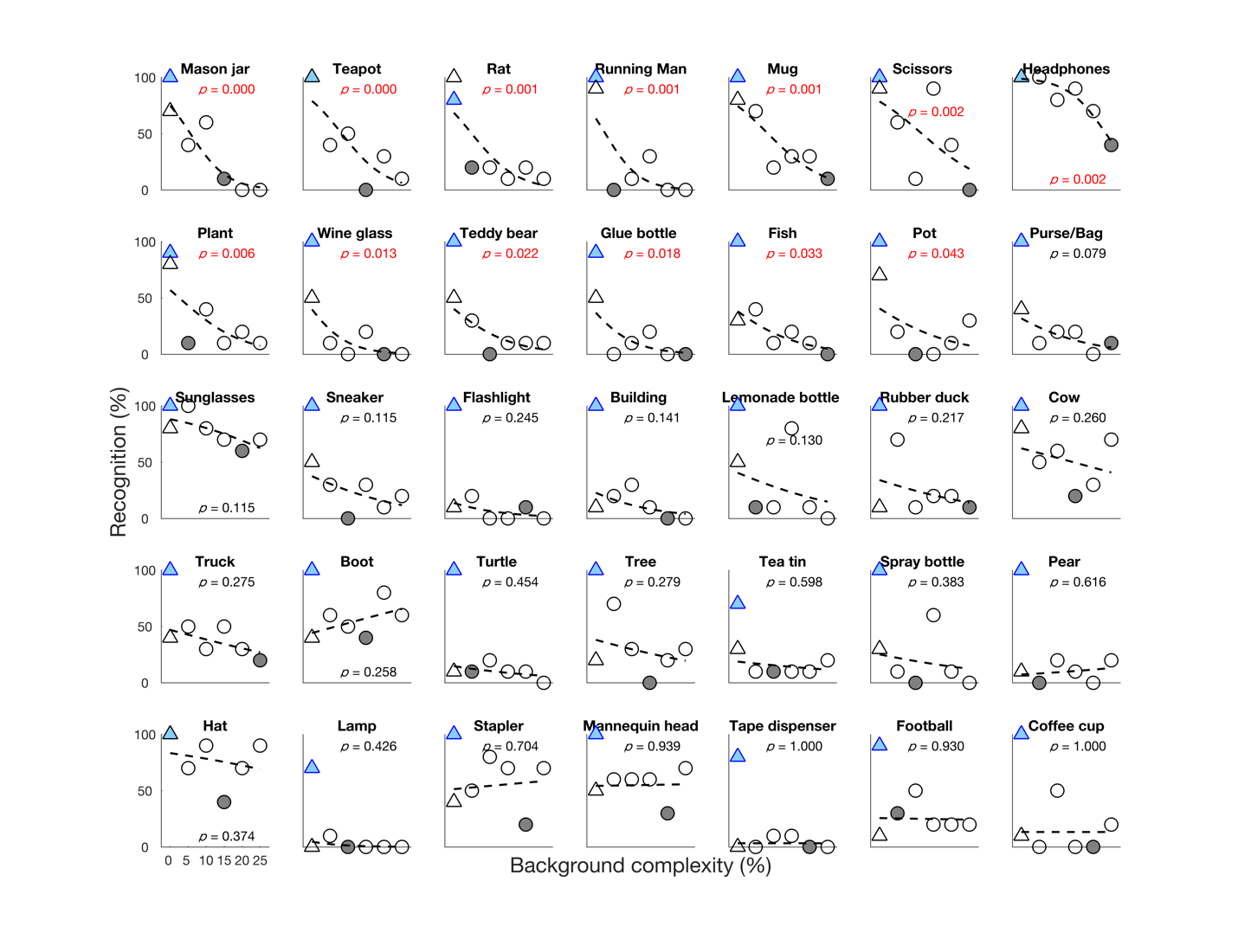 |
| --- |
| Figure A1. Recognition rate of each individual object and background complexity. The blue filled triangles represent the recognition rates of the high-resolution, no background control images. The black symbols show the performance of the simulated prosthetic vision conditions, among which the black triangles indicate the condition without clutter and black circles for conditions with clutter. The filled black circles correspond to background complexity selected for the Motion Parallax experiment. The black dashed lines are logistic fits for the recognition as a function of background complexity levels. The *p* values (the Wald test) show whether the coefficients of the background complexity in the logistic regression model are significantly different from 0, and thus represent the effect of background on object recognition. Significant effects are noted in red and the objects are ordered according to the background effect (increasing *p* values). |

Figure A2 represents recognition rates calculated based on 10 subjects’ responses for each object in each condition in the Motion Parallax experiment. Given the non-normality observed in the data, we used a nonparametric test, the Wilcoxon signed-rank test, for statistical analysis.

| 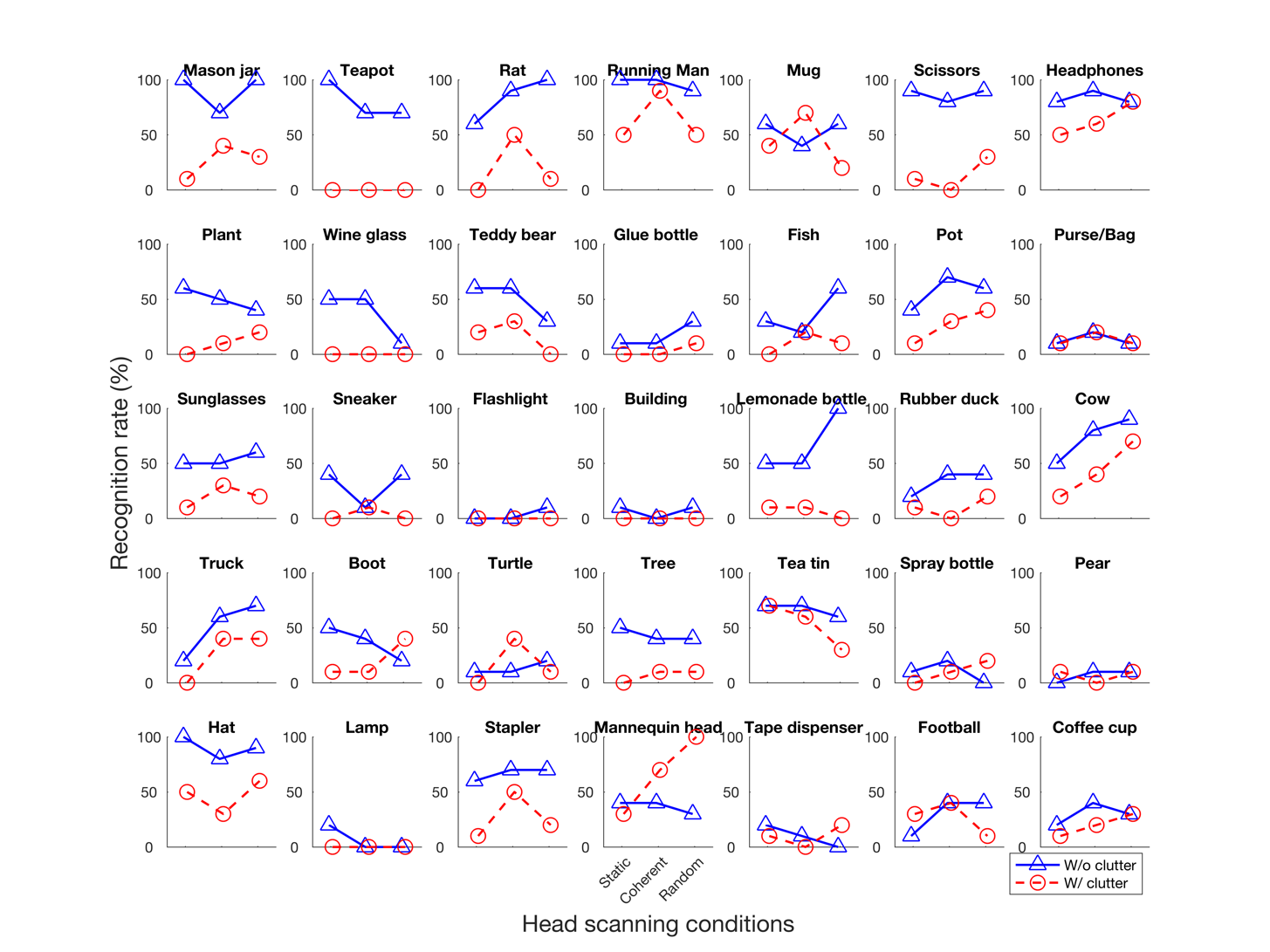 |
| --- |
| Figure A2. Recognition rates of each object by condition. Ten responses collected for each object from 10 randomly selected subjects under each experimental condition were used to calculate the recognition rate. Blue triangles and solid lines represent conditions without background clutter, and red circles and dashed lines are conditions with clutter. The three scanning conditions are displayed along the x-axis: static, coherent, and random multiple viewpoints. |

## Alternative analysis of recognition rates in the Motion Parallax experiment

In the main text, the recognition rate for each image under each condition were computed across subjects. Alternatively, the recognition rate can be calculated for each subject across images; that is, we calculated the recognition rate as the ratio of the number of correctly recognized images over the total number of images for that subject and condition. A two-way repeated measures ANOVA (background and scanning conditions as the within-subject factors) was conducted. The data did not violate the sphericity assumption by the Mauchly’s test (for the factor of scanning condition, test statistic $W= 0.99, p=0.90$; for the interaction term, $W= 0.97, p=0.52$). Similarly, as reported in the main text, both the background ($F\left( 1,59 \right)=157.6, p<0.0001$) and the scanning condition ($F\left( 2,118 \right)=5.39, p=0.0058$) factors significantly affected the recognition rates. A weak interaction was also found between the background and scanning conditions ($F\left( 2,118 \right)=3.52, p=0.033$).

## Results of head movement analysis in the Motion Parallax experiment

The displayed image sequence for each trial indicated quantized head positions, and the number of unique images was used to measure the range of subjects’ head movement. Figure A3 shows the median number of unique displayed images for each condition. The static and no clutter condition had the smallest median frames, 4.8, and the median number of frames of the coherent with clutter condition was the largest, 5.8. More frames were explored in the conditions with clutter than without (the Wilcoxon signed-rank test approximate $z=3.66, p=0.0001$, symmetry test *p* = 0.55). The coherent ($z=4.77, p<0.0001$, symmetry test *p* = 0.74) and random scanning ($z=3.13, p=0.0009$, symmetry test *p* = 0.40) conditions both had significant more unique frames than the static condition. With clutter, more unique frames were seen while coherent scanning than the static condition ($z=3.81, p=0.0001$, symmetry test *p* = 0.34).

| 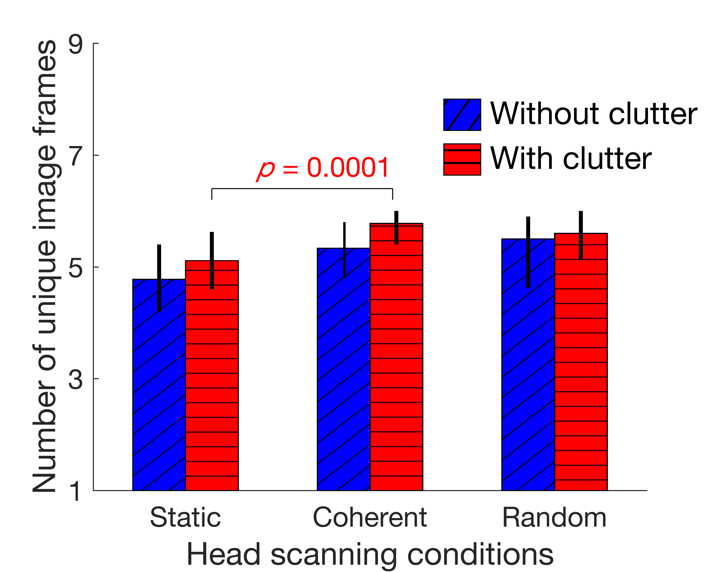 | Figure A3. The median number of unique image frames viewed for each condition across the 35 objects (averaged across subjects). Slightly but significantly more frames were explored in the conditions with clutter, and the conditions with coherent or random scanning. Error bars represent the interquartile range. |
| --- | --- |

Fourier analysis was performed on the quantized head position signals to determine the main head scanning frequency in cycles per minute. To find the head movement frequency for each trial, the quantized head positions as a function of time were Fourier transformed into the frequency domain (Fig. A4). The frequency with the highest amplitude was selected as the main head scanning frequency. Time for each cycle of movement was calculated from the main frequency. Across all subjects and trials, the median main scanning frequency was about 6 cycles/min, which means that it took about 10 s on average for subjects to complete one scanning cycle.

| 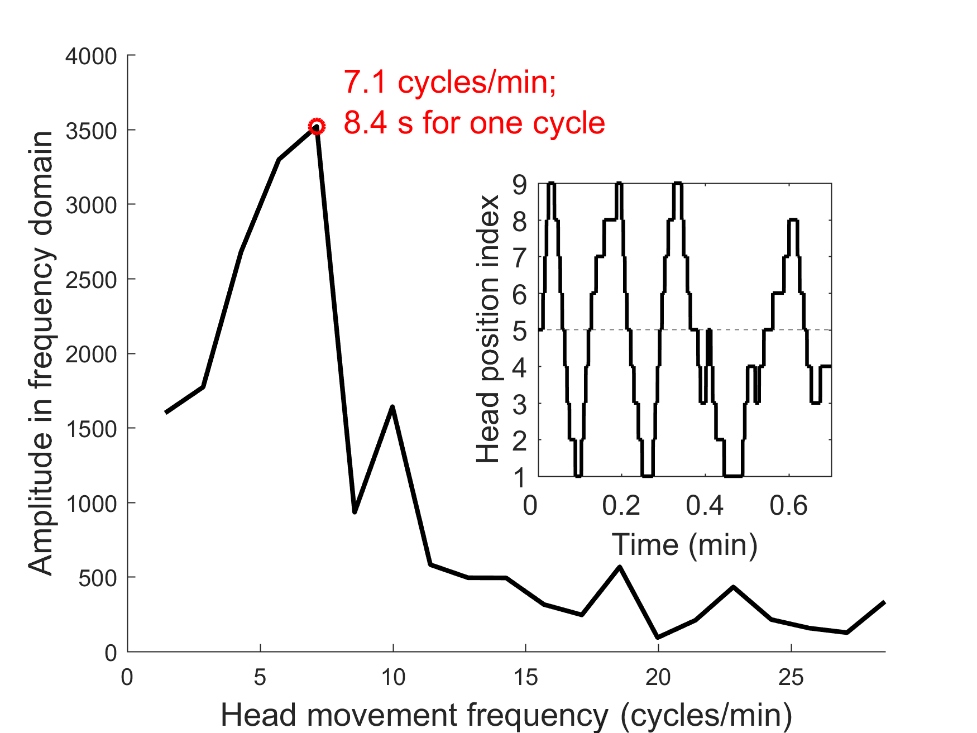 | Figure A4. Fourier analysis of the quantized head position sequences (as shown in inset). The frequency with the highest amplitude was taken as the main head scanning frequency (red circle). The DC component is not shown. |
| --- | --- |
